# Association between serum uric acid to high-density lipoprotein ratio and all-cause in hypertensive patients: Mediating role of neutrophils

**DOI:** 10.1371/journal.pone.0325620

**Published:** 2025-06-05

**Authors:** Chunyu Yan, Yabin Zhou, He Wang, Jiamei Fu, Qian Xu

**Affiliations:** 1 Department of Cardiology, The First Hospital Affiliated to Heilongjiang University of Traditional Chinese Medicine, Harbin, People’s Republic of China; 2 Department of Cardiology, The Second Hospital Affiliated to Heilongjiang University of Traditional Chinese Medicine, Harbin, People’s Republic of China; Central University of Andhra Pradesh, INDIA

## Abstract

**Background:**

The aim of this study was mainly to investigate the association between Serum uric acid (SUA) to high-density lipoprotein cholesterol (HDL-C) ratio (UHR) and all-cause mortality in hypertensive patients,and to further investigate the mediating role of neutrophils.

**Methods:**

Our cohort study included 4533 hypertensive patients drawn from the 2005–2018 National Health and Nutrition Examination Survey (NHANES) database and combined with the National Death Index (NDI) database to obtain mortality data for subjects. Kaplan-Meier survival curves, multifactorial Cox risk-proportional modeling, restricted cubic spline (RCS)-based smoothed curve fitting, threshold effects analysis, and subgroup analyses were performed to evaluate the associations between UHR and all-cause mortality, and, finally,causal mediating effects were performed to analyze the mediating role of neutrophils.

**Results:**

Over a mean duration of 90.32 months, the follow-up all-cause mortality occurred in 1003 individuals, and the mean age of all subjects included was (61.69 ± 14.28) years, and the Kaplan-Meier survival curves demonstrated that high levels of UHR were notably connected to lower survival. In multivariate Cox regression analysis, high quartile UHR was positively connected to all-cause mortality (HR: 1.36, 95% CI: 1.03,1.80, *P* = 0.031), and smoothed curve fitting combined with threshold effect analysis showed a nonlinear relationship between UHR and all-cause mortality, with a curve inflection point of 0.14, i.e., when UHR < 0.14, an increase in UHR did not affect the increase in all-cause mortality (HR: 0.84, 95% CI: 0.06, 11.51, *P* = 0.8968), and when UHR > 0.14, the all-cause mortality increased with the increase in UHR. We further stratify by gender and find that the inflection point for male UHR is 0.13, the suggesting that the association between UHR and all-cause mortality increased with increasing UHR when UHR was < 0.13, HR (95% CI): 0.01 (0.00, 0.22), *P* < 0.01 and when UHR > 0.13, HR (95% CI): 0.41 (0.04, 1.36), *P* < 0.01. However there was a significant linear correlation for females (HR: 1.31 95% CI: 0.15, 11.55, *P* < 0.0001). Analysis of causal mediating effects elucidated that the proportion of neutrophils mediating the association between UHR and all-cause mortality was 18.63%.

**Conclusion:**

There was a significant positive correlation between elevated UHR and all-cause mortality in hypertensive patients, and this association may be mediated with neutrophils.

## Introduction

In its report on the global devastation of hypertension, the World Health Organization (WHO) suggested that hypertension affects the health of one-third of the world’s adult population as a causative factor in many diseases, including cardiovascular disease, stroke, and renal failure, and that a significant public health concern in both rich and economically developing nations is hypertension [1,2].In addition, relevant studies have confirmed that hypertension is also an cancer treatment and prognosis [3]. At this stage, there is often poor treatment in people with hypertension, which ultimately leads to death or disability; therefore, it is urgent to explore the causes and mechanisms of hypertension from multiple aspects and angles for its prevention and treatment.

UHR is a novel biomarker that has the ability to better reflect inflammatory factors and metabolic-related diseases (e.g., acute coronary syndrome, metabolic syndrome, type 2 diabetes mellitus, insulin resistance, and Hashimoto’s thyroiditis) than SUA and HDL-C alone [4,5,6,7]. In humans, SUA is a metabolite of purines that is typically eliminated by the kidneys [8], and studies have shown that increased levels of SUA may induce hypertension through activation of the renin-angiotensin-aldosterone system (RAAS), leading to endothelial dysfunction and vasoconstriction, further increasing sympathetic arousal, and inducing inflammatory responses [9,10,11,12,13]. Prior research has indicated that SUA has a separate pathophysiological function in the onset of hypertension and hypertension-associated cardiovascular events, and that elevated serum uric acid not only leads to lipid metabolism disorders, but also is strongly associated with all-cause mortality and cardiovascular mortality [14,15]. Weifang Liu et al. analyzed the potential effects of lipids, lipoprotein particles, and circulating metabolites on systolic and diastolic blood pressure by the MR Bayesian model averaging method (MR-BMA), and found that the strongest correlations were found between TG, HDL-C, and increases in SBP and DBP [16]. Although G. Aktas et al. found that increased UHR levels were closely linked to blood pressure fluctuations [17], there is a lack of survival analysis of the prognosis of hypertensive patients, and in addition, since inflammatory factors are crucial in the development of high blood pressure and in the prediction of all-cause mortality [18,19], we believe that neutrophils may have a prognostic role to assess the prognosis of hypertensive patients.

In summary, we conducted a prospective cohort study on the association between UHR and all-cause and cardiovascular mortality and further evaluated the function of neutrophils as mediators.

## Methods and materials

### Study population and design

**Fig 1:**
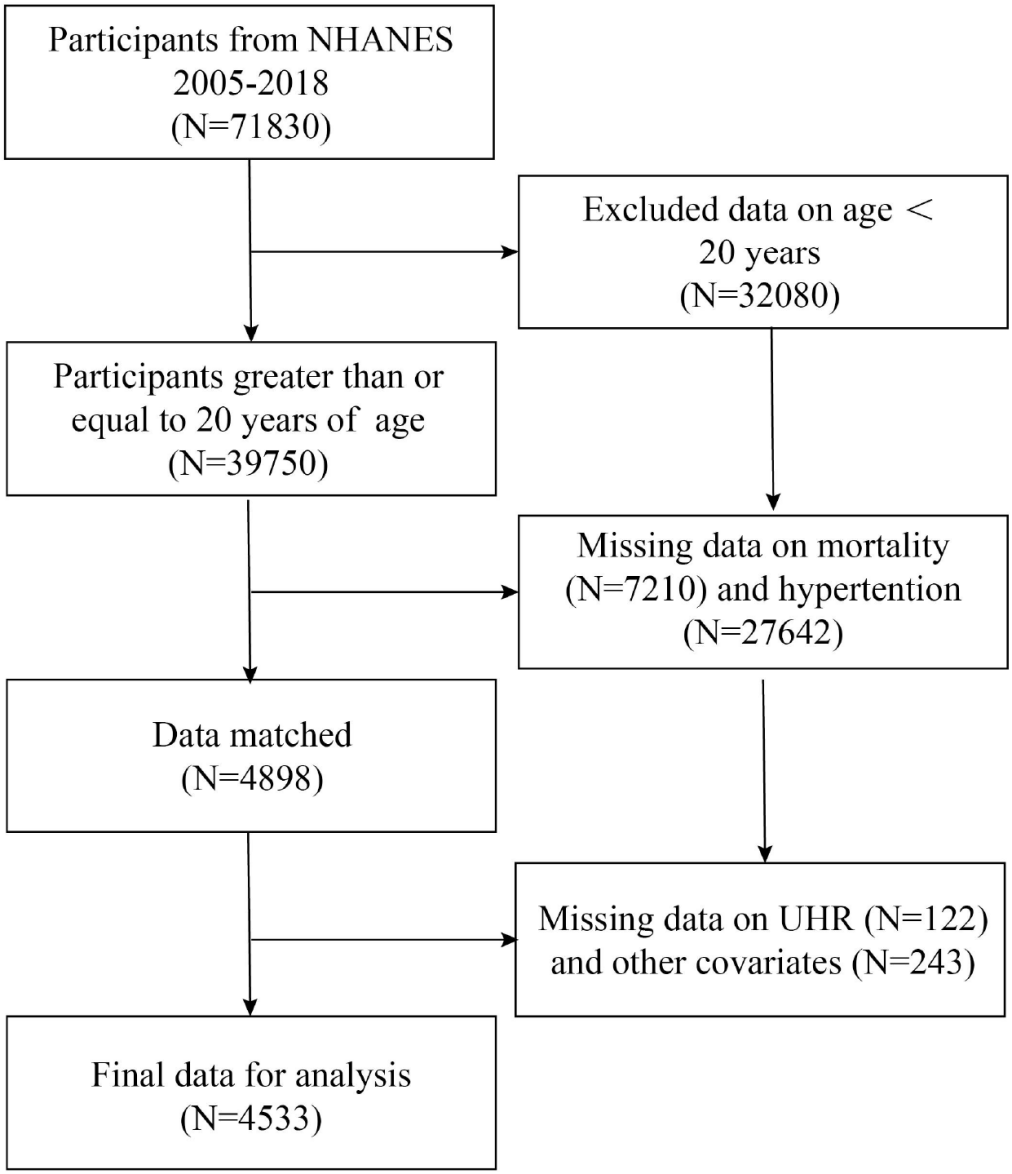
Flowchart of study participants.

The NHANES database provided the data that were chosen for this long-term cohort study.To guarantee that the study sample is representative, NHANES uses a sophisticated sampling design [20]. The NHANES database is a priceless tool that offers crucial information to comprehend and enhance the nutritional and health condition of the American people. It also aids in the investigation of illness risk factors and causal variables by scientists [21]. The National Center for Health Statistics’ Institutional Review Board approved the NHANES cohort study, and each participant gave written informed permission [22]. In the current prospective cohort study, we used data from the 2005–2018 NHANES cycle involving a total of 71,830 subjects after excluding age younger than 20 years (N = 32080), missing mortality (N = 7210), hypertension (N = 27642),UHR (N = 122), drinking status (N = 143), smoking status (N = 48), Neutrophil (N = 12), and finally 4533 subjects were included in the study, and the flow chart is as follows (Fig 1).

### Exposure variable

Our study used UHR as a continuous type of exposure variable, and UHR was calculated as SUA divided by HDL-C. Hypertension was defined according to the new guidelines for hypertension established by the American Heart Association, i.e., a physician's report of the diagnosis, use of hypertension medication, and a systolic blood pressure ≥80 or/and a diastolic blood pressure ≥130 mmHg [23]. The included hypertensive patients were analyzed in quartiles based on UHR values.

### Outcome variable

The primary outcome of our study was all-cause mortality and the secondary outcome was cardiac cause mortality, and mortality data in NHANES were obtained by correlating with the National Death Index Database (NDI) (https://www.cdc.gov/nchs/data-linkage/mortality-public.htm) as of December 31, 2019 records were correlated to determine this. We assessed all-cause mortality, as well as mortality due to cardiovascular diseases (I09-I11, I13, I20, I51-I60, I69-I00) using the International Classification of Diseases and Related Health Problems, Tenth Revision [24].

### Covariates

Our covariates for this study included gender, age, household poverty-to-income ratio (PIR), race, education, marital status, smoking status (never smokers, current smokers, and ex-smokers), alcohol consumption, body mass index (BMI), HDL-C, total cholesterol (TC), triglycerides (TG), SUA, neutrophils, platelets, heart failure, coronary heart disease (CHD), and diabetes. Alcohol consumption was categorized into two groups based on the questionnaire, “Did the participant drink at least 12 glasses of wine/1 year” (ALQ101), with 1 unit of beverage equivalent to 12 ounces of beer, 5 ounces of wine, or 1.5 ounces of liquor.

### Statistical analysis

To make the results of the study more reliable, we used the weighting procedure recommended by NHANES for weighting specific groups. The mean ± standard deviation was used to express continuous variables, whereas percentages were used to express categorical variables.To assess the between-group variability, the chi-square test or Kruskal-Wallis H test was performed on the interquartile range UHR, and P < 0.05 indicated that the statistical results were significant.

Cox proportional risk model were used to estimate the association between UHR and all-cause mortality and cardiovascular disease mortality, expressed as mortality risk ratios (HR) and 95% confidence intervals (CI). We adjusted the different covariates separately for weighted analysis, model 1 was not adjusted for any covariates, model 2 was adjusted for age, sex, and race, and model 3 (fully adjusted model) was adjusted for age, sex, race, smoking, alcohol consumption, BMI, TC, heart failure,CHD, diabetes, Neutrophils, and Platelets, using the Kaplan-Meier survival curves to plot the association between time and survival, and compared using Log-rank test. Cox regression analyses were performed using smooth curve fitting based on the Restricted Cubic Spline Analysis (RCS) algorithm, and turning points were explored using threshold effects analysis for nonlinear associations, we further stratified our analysis by gender. In addition, we performed subgroup analyses of the association between UHR and all-cause and cardiovascular disease mortality using fully adjusted models, and finally, the “mediation” package and the “probit” probability function in R 4.2.2 were used to analyze the causal mediating effects of neutrophils, and to assess the overall, direct, and indirect effects of neutrophils.

### Ethics declarations

Any study involving human subjects was reviewed and approved by the NCHS Research Ethics Review Board. To participate in the study, each participant's legal guardian or next of kin gave their written informed consent.

## Results

### Characteristics of respondents

Of the 4533 respondents enrolled, the mean age was (61.69 ± 14.28) years, with 53.14% male and 46.86% female. We quartile the UHR levels as QI (0.01–0.08, N = 1132), Q2 (0.08–0.11, N = 1133), Q3 (0.11–0.15, N = 1131), and Q4 (0.15–0.56, N = 1137).In contrast with Q1, Q4 had an increase in the number of males, BMI, alcohol consumption, TG, Neutrophil, higher education level, increased prevalence of CHD and heart failure, and decreased smoking, TG, HDL-C. Age, gender, race, education level, marital status, smoking, alcohol consumption, BMI, HDL-C, TC, TG, Uric acid, Neutrophil, Heart failure, and CHD were all significantly different between the four groups.The results are shown in Table 1.

**Table 1:** Characteristics of study populations with different UHR quartiles.

Characteristics	Overall (N = 4533)	Q1 (N = 1132)	Q2 (N = 1133)	Q3 (N = 1131)	Q4 (N = 1137)	*P*-value
GENDER						<0.001
Male	2409(53.14%)	303 (26.77%)	515 (45.45%)	710 (62.78%)	881 (77.48%)	
Female	2124(46.86%)	829 (73.23%)	618 (54.55%)	421 (37.22%)	256 (22.52%)	
Age,years	61.69 ± 14.28	64.52 ± 13.14	62.71 ± 13.96	61.21 ± 14.28	58.31 ± 15.75	<0.001
PIR	2.03 ± 1.56	2.40 ± 1.56	2.35 ± 1.55	2.37 ± 1.56	2.37 ± 1.55	0.936
Race, n (%)						<0.001
Mexican American	624(13.77%)	153 (13.52%)	167 (14.74%)	162 (14.32%)	142 (12.49%)	
Other Hispanic	395(8.69%)	76 (6.71%)	109 (9.62%)	110 (9.73%)	100 (8.80%)	
Non-Hispanic White	1906(42.05%)	506 (44.70%)	415 (36.63%)	465 (41.11%)	520 (45.73%)	
Non-Hispanic Black	1219(26.89%)	315 (27.83%)	335 (29.57%)	298 (26.35%)	271 (23.83%)	
Other Race	389(8.6%)	82 (7.24%)	107 (9.44%)	96 (8.49%)	104 (9.15%)	
Education level, n (%)						0.035
Below high school	730(16.10%)	162 (14.31%)	188 (16.59%)	203 (17.95%)	177 (15.57%)	
High school	744(16.41%)	173 (15.28%)	212 (18.71%)	168 (14.85%)	191 (16.80%)	
Above high school	3059(67.49%)	797(70.41%)	733(64.7%)	760(67.20%)	769(67.64%)	
Marital status,n(%)						<0.001
Married	2367(52.22%)	523 (46.20%)	571 (50.40%)	629 (55.61%)	644 (56.64%)	
Widowed	732(16.15%)	241 (21.29%)	215 (18.98%)	152 (13.44%)	124 (10.91%)	
Divorced	589(12.99%)	168 (14.84%)	143 (12.62%)	146 (12.91%)	132 (11.61%)	
Separated	153(3.38%)	47 (4.15%)	37 (3.27%)	37 (3.27%)	32 (2.81%)	
Never married	474(10.46%)	109 (9.63%)	115 (10.15%)	119 (10.52%)	131 (11.52%)	
Living with partner	218(4.80%)	44 (3.89%)	52 (4.59%)	48 (4.24%)	74 (6.51%)	
Smoking,n(%)						<0.001
Never	1359(29.98%)	413(40.29%)	489(42.68%)	457(42.03%)	532(46.95%)	
Former	1984(43.77%)	568(50.21%)	513(45.37%)	491(43.61%)	412(40.28%)	
Now	1190(26.25%)	367(36.79%)	216(25.37%)	319(31.64%)	288(29.18%)	
Drinking, n (%)						<0.001
<12 drinks/year	1679(37.04%)	379(37.15%)	518(46.01%)	459(41.97%)	323(32.04%)	
≥12 drinks/year	2854(62.96%)	671(59.27%)	751(65.76%)	833(74.42%)	599(52.89%)	
BMI,kg/m2	29.85 ± 6.69	26.54 ± 5.88	29.74 ± 6.90	30.74 ± 6.93	32.39 ± 7.03	<0.001
HDL-C, mmol/L	55.34 ± 11.28	73.20 ± 17.74	56.39 ± 10.82	47.57 ± 8.15	37.90 ± 7.57	<0.001
TC, mmol/L	198.85 ± 44.71	207.87 ± 41.93	199.08 ± 39.93	200.34 ± 45.41	197.17 ± 48.47	<0.001
TG, mmol/L	148.24 ± 102.73	100.01 ± 57.23	120.37 ± 65.33	149.42 ± 106.13	218.31 ± 204.29	<0.001
Uric acid,μmol/L	5.57 ± 1.14	4.43 ± 1.02	5.39 ± 1.01	6.07 ± 1.04	7.17 ± 1.29	<0.001
Neutrophil, 10^9^/L	4.28 ± 1.65	3.92 ± 1.57	4.16 ± 1.63	4.27 ± 1.58	4.68 ± 1.79	<0.001
Platelets, 10^9^/L	243.52 ± 67.84	245.14 ± 69.59	242.78 ± 66.22	241.01 ± 66.98	238.22 ± 66.05	0.094
Heart failure, n (%)	229(5.05%)	40 (3.53%)	42 (3.71%)	70 (6.19%)	77 (6.77%)	0.002
CHD, n (%)	298(6.57%)	43 (3.80%)	71 (6.27%)	79 (6.98%)	105 (9.23%)	<0.001
Diabetes, n (%)	916(20.21%)	204 (18.02%)	228 (20.12%)	236 (20.87%)	248 (21.81%)	0.140

Abbreviations: *UHR* serum uric acid to HDL-cholesterol ratio, *PIR* poverty-to-income ratio, *BMI* body mass index, *TC* total cholesterol, *TG* triglycerides, *HDL-C* HDL-cholesterol, *CHD* Coronary heart disease.

### Association of UHR with all-cause and cardiovascular mortality in hypertensive patients

**Fig 2:**
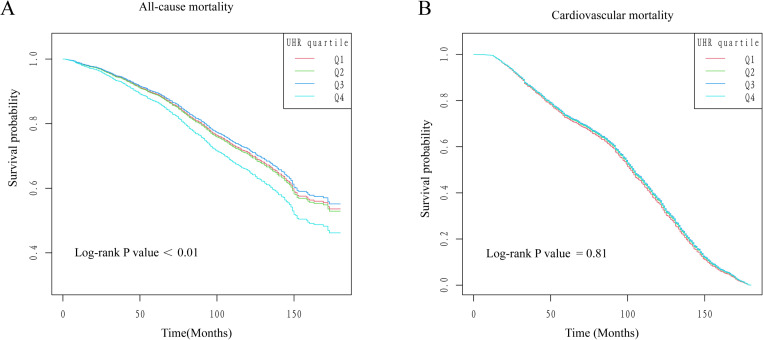
Kaplan-Meier curves for UHR quartile classification (A: all-cause mortality, B: cardiovascular mortality).

At a median follow-up of 90.32 months, all-cause mortality occurred in 1,003 individuals, with cardiovascular mortality occurring in 272 individuals.As shown in Table 2, when UHR was treated as a continuous variable, in Model 3, we found that for every 1-unit rise in UHR, the probability of having an all-cause death increased by 11%, when the UHR is converted to a categorical variable (interquartile range), in Model 3, Q4 compared with Q1, we found that for every 1-unit rise in UHR, the probability of an all-cause death increased by 42%, and the difference was significant, whereas in none of the three adjusted models did we find a noteworthy correlation between UHR and cardiovascular mortality. In addition, the results of the Kaplan-Meier survival curve analysis based on UHR quartiles are shown in Fig 2, Q4 had a markedly higher all-cause mortality rate than the other three groups (P < 0.01), however, there was not a discernible distinction between UHR quartiles (Q1-Q4) in terms of cardiovascular mortality(P = 0.81).

**Table 2:** Association of UHR with all-cause and cardiovascular mortality in hypertensive patients.

Adjusted HR (95% CI),*P* Value			
UHR,mg/dL	Model 1	Model 2	Model 3
All-cause mortality			
Continuous quartiles	1.01(0.92,1.09),0.947	1.12(1.03,1.23),0.012	1.11(1.01,1.21),0.04
Q1	Ref.	Ref.	Ref.
Q2	1.02(0.80,1.31),0.845	1.06(0.84,1.35),0.598	1.04(0.81,1.34),0.749
Q3	0.89(0.69,1.14),0.346	1.04(0.80,1.36),0.745	1.02(0.77,1.34),0.917
Q4	1.05(0.82,1.35),0.676	1.48(1.13,1.93),0.005	1.36(1.03,1.80),0.031
*P* for trend	0.947	0.012	0.048
CVD mortality			
Continuous quartiles	1.37(0.65,2.92),0.407	1.55(0.63,3.79),0.338	0.91(0.34,2.44),0.856
Q1	Ref.	Ref.	Ref.
Q2	1.08(0.94,1.24),0.260	1.05(0.92,1.20),0.438	1.04(0.91,1.18),0.586
Q3	1.01(0.90,1.13),0.881	0.99(0.90,1.10),0.916	0.98(0.88,1.11),0.787
Q4	1.07(0.95,1.21),0.241	1.08(0.94,1.24),0.274	0.99(0.86,1.16),0.99
*P* for trend	0.418	0.407	0.820

Model 1 adjust for: none. Model 2 adjust for: gender, age, race. Model 3 adjust for: gender, age, race, smoking, drinking, BMI,TC,heart failure,CHD,diabetes,Neutrophils,Platelets.

**Fig 3:**
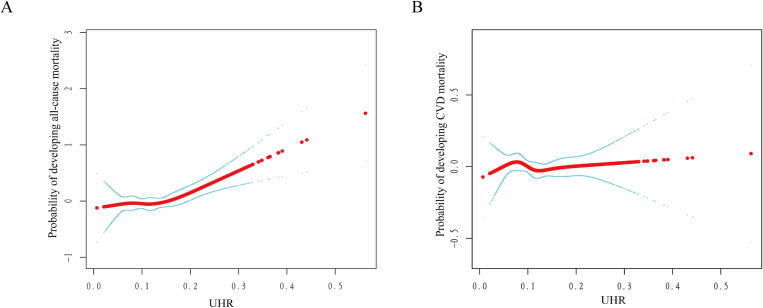
Dose relationship between UHR and all-cause mortality. (A) and cardiac cause mortality (B) in patients with hypertension, where the solid red line represents a smooth curve fit between the variables and the blue bar indicates the 95% confidence interval of the fitted curve.

Further, we used smoothed curve fitting based on restricted cubic spline (RCS) analysis (Fig 3) as well as threshold effects analysis (Table 3), which revealed a nonlinear association between UHR and all-cause mortality in a multifactorial Cox risk model, and the threshold effect analysis showed an inflection point of 0.14, no greater risk of death from all causes as UHR rises when UHR < 0.14 (HR: 0.84, 95% CI: 0.06, 11.51, *P* = 0.897), and when UHR > 0.14, the likelihood of incidence of all-cause mortality tended to increase significantly with increasing UHR (HR: 41.56, 95% CI: 6.58, 262.30, *P* < 0.0001), but there was not a discernible distinction between UHR and incidence of cardiovascular mortality (HR: 0.97, 95% CI: 0.48–1.96, *P* = 0.936).

**Table 3:** Analysis of the threshold effect of UHR on all-cause mortality and cardiac cause mortality in hypertensive patients.

Adjusted HR (95% CI), *P* Value	
All-cause mortality	
Model 1	
Fitting by the standard linear model	9.18 (2.48, 33.95),0.0009
Fitting by the two-piecewise linear model	
Breaking point(K)	0.14
<K-band effect 1	0.84 (0.06, 11.51),0.8968
>K-band effect 2	2.56 (1.58, 16.31), < 0.0001
Difference in effect between 2 and 1	4.94 (1.25, 19.54),0.0376
*P* for log likelihood ratio test	0.041
CVD mortality	
Model 1	
Fitting by the standard linear model	0.97 (0.48, 1.96), 0.9360
Fitting by the two-piecewise linear model	
Breaking point(K)	0.2
<K-band effect 1	0.67 (0.28, 1.62),0.3764
>K-band effect 2	3.56 (0.51, 15.07), 0.2020
Difference in effect between 2 and 1	5.31 (0.49, 17.01),0.1684
*P* for log likelihood ratio test	0.176

### Association between UHR and all-cause mortality stratified by sex

**Fig 4:**
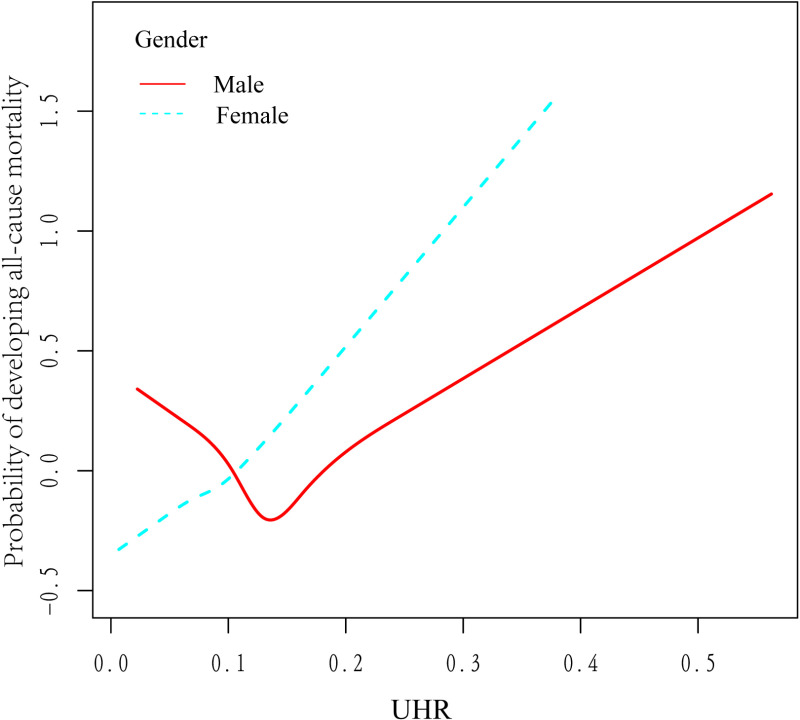
Dose-response relationship between UHR and probability of all-cause mortality stratified by sex.

Based on previous literature, we found that gender emerged as a strong confounder of the connection between UHR and all-cause mortality; therefore, Our smoothed curve fitting (Fig 4) combined with threshold effect analysis (Table 4), stratified by sex, showed that males had a U-shaped association between UHR and all-cause mortality, with an inflection point of 0.13, indicating that HR (95% CI): 0.01 (0.00, 0.22), *P* < 0.01, when UHR < 0.13, and that HR (95% CI):0.41 (0.04, 1.36), *P* < 0.01, when UHR > 0.13. However, females did not have a nonlinear relationship (log likelihood ratio test *P* = 0.373) but a significant linear association (HR (95% CI): 1.31 (0.15, 11.55), *P* < 0.0001).

**Table 4:** Threshold effect analysis of UHR and all-cause mortality in hypertensive patients stratified by sex.

Adjusted HR (95% CI), *P* Value	
Male	
Model 1	
Fitting by the standard linear model	2.02 (0.37, 11.14),0.4206
Fitting by the two-piecewise linear model	
Breaking point(K)	0.13
<K-band effect 1	0.01 (0.00, 0.22),0.0056
>K-band effect 2	0.41(0.04, 1.36), 0.0008
Difference in effect between 2 and 1	7.14 (0.53, 9.52), 0.0004
*P* for log likelihood ratio test	<0.001
Female	
Model 1	
Fitting by the standard linear model	1.31 (0.15, 11.55) <0.0001
Fitting by the two-piecewise linear model	
Breaking point(K)	0.18
<K-band effect 1	0.56 (0.03, 9.87),0.0059
>K-band effect 2	1.53 (0.58, 14.05),0.0099
Difference in effect between 2 and 1	0.72 (0.02, 3.36),0.3618
*P* for log likelihood ratio test	0.373

### Subgroup analysis

**Fig 5:**
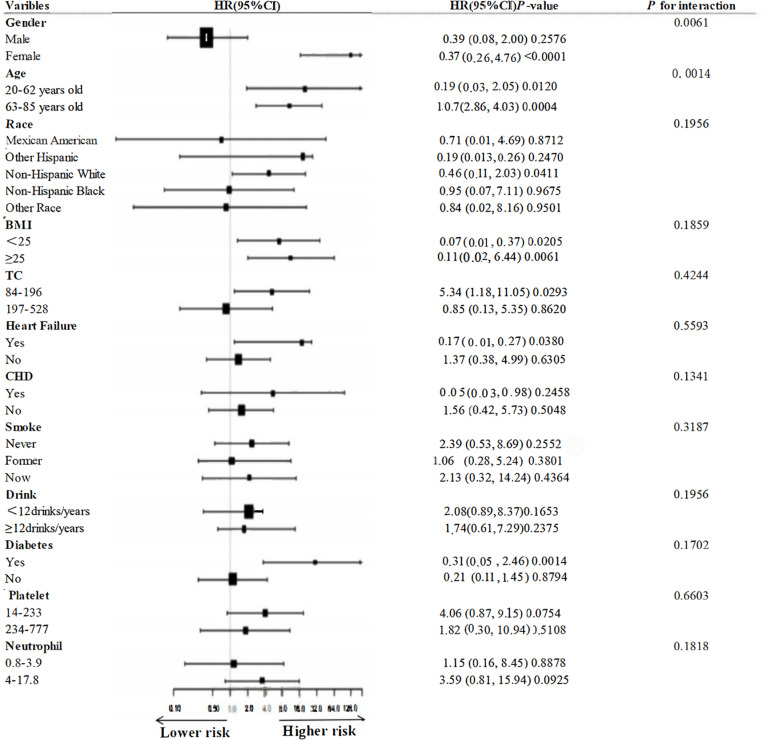
Subgroup analysis of the association between UHR and all-cause mortality.

**Fig 6:**
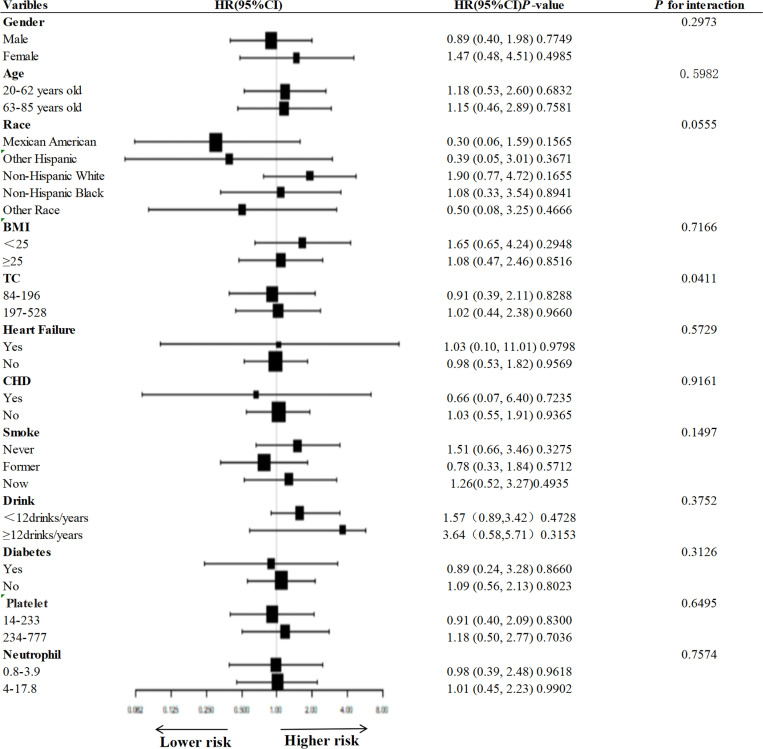
Subgroup analysis of the association between UHR and cardiovascular mortality.

Figs 5 and 6 demonstrate subgroup analyses based on sex, age, race, BMI, TC, smoking, alcohol consumption, heart failure, CHD, diabetes mellitus, platelets, and neutrophils, and in the vast majority of subgroups, UHR was not associated with all-cause and cardiovascular mortality, with the exception of sex, and age, which were significantly different between UHR and all-cause mortality (interaction test *P* < 0.01) significant differences (interaction test *P* > 0.05).

### Mediating effect analysis of neutrophils

**Fig 7:**
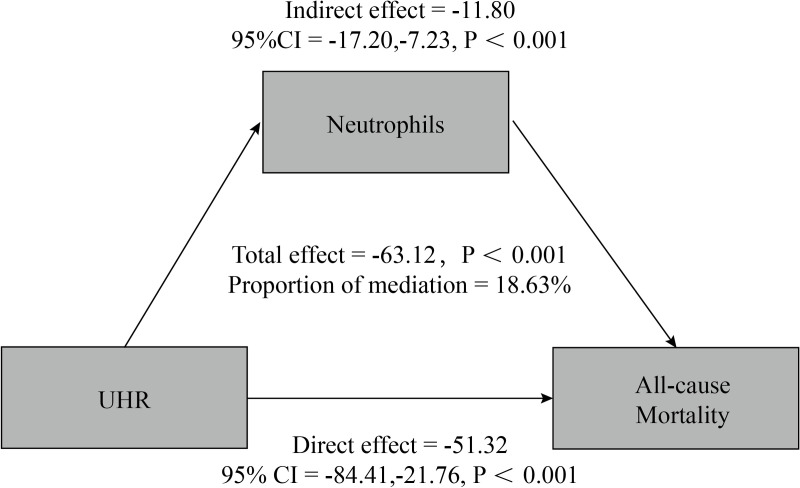
Correlation analysis between neutrophil-mediated UHR and all-cause mortality rate.

Fig 7 shows that neutrophils mediate the relationship between UHR and all-cause mortality, with a mediating effect ratio of 18.63.

## Discussion

This is the first larger prospective study that we are aware of on the relationship between UHR levels and all-cause mortality and CVD mortality in hypertensive patients. In this cohort study, a U-shaped correlation was discovered between UHR and all-cause mortality in hypertensive patients. Stratified by gender, we found that there was a U-shaped relationship between UHR level and all-cause mortality in male hypertensive patients, and analysis of the threshold effect showed that all-cause mortality was lowest at a UHR level of 0.14 in men, and that all-cause mortality in female hypertensive patients increased with increasing UHR level, and the two were linearly associated. followed by an increase, and the two were linearly correlated. Causal mediation analysis emphasized the important role of neutrophils in the association between UHR and all-cause mortality, suggesting that inflammation may be a potential mechanism for the association. Therefore, our study suggests that UHR may be an effective biomarker for health management in hypertensive populations.

UHR is a relatively novel biomarker calculated by dividing SUA level by HDL-C. In particular, in humans, endogenous and exogenous purine metabolism result in uric acid (UA), of which the kidneys eliminate two thirds [25]. Elevated UA is not only a separate risk element for hypertension, but also a major cause of the high incidence of cardiovascular disease. Elevated UA adversely affects the vascular system, leading to endothelial damage and triggering an inflammatory response [26]. In addition, UA scavenges oxygen free radicals and functions as an antioxidant, and elevated levels of UA cause oxidative stress and the production of free radicals, which increases the risk of developing hypertension [27,28]. Numerous clinical reports have shown the connection between UA and hypertension.Zulkarnain Mustapha et al. in a case-control analysis of blood samples collected from 132 subjects (88 hypertensive patients as a case group and 44 subjects as a control group) found that for every 1-unit increase in SUA, there was an increase in the probability of hypertension by a factor of 1.005 (P < 0.05) [29]. In addition, Lihua Hu et al. found that both low and high concentrations of SUA were associated with increased mortality from all-cause, cardiovascular and respiratory diseases through a median follow-up of 9118 adults over 5.83 years [30]. In addition, disorders of lipid metabolism cause lipid particles to be deposited in the vessel wall which can lead to endothelial damage, endothelial dysfunction and formation of atherosclerotic plaques, and reduced vascular elastic compliance leading to abnormal blood pressure regulation [31]. Dyslipidemia is not only a causative factor of hypertension, but also a major driver of cardiovascular events and mortality in hypertensive patients [32].Many previous studies have consistently demonstrated a negative relationship between HDL-C and hypertension [33,34,35]. However, it has also been found that as HDL-C levels become too high they instead lose their protective effect on the vascular endothelium, when the structure and function of HDL-C are altered thereby adversely affecting blood pressure [36,37]. A number of prospective investigations have recently examined the relationship between HDL-C and mortality and cardiovascular events in people with hypertension.Rufei Liu et al. in a Systolic Blood Pressure Intervention Trial of 9323 hypertensive patients found that patients with hypertension who had HDL-C levels over 80 mg/dL were at a decreased risk of dying from cardiovascular disease [31]. Valentina Trimarco et al. found a U-shaped association between HDL-C and the risk of cardiovascular events in 11,987 hypertensive patients followed for a mean of (25.1 ± 31.2) months [38], and in addition, higher HDL-C levels were linked to a decreased incidence of MACE in males but not in females among elderly hypertension patients [39]. The combination of uric acid and HDL-C is as a more sensitive marker of metabolic and inflammatory status than either of the single indicators [40].Gulali Aktasa et al. analyzed a cross-sectional cohort study and found that for every unit increase in UHR, the risk of poor blood pressure control increased by 7.3-fold [17].Rana Kolahi Ahari et al. observed 9704 participants aged between 35–65 years and found that UHR levels were higher in men than in women in the group of hypertensive patients [41]. However there is a lack of analysis on UHR and prognosis in hypertensive patients and our study fills this gap.

Our present study developed three models to explore the association of UHR between all-cause mortality and cardiovascular mortality in hypertensive patients. The findings showed that the highest quartile of UHR was associated with increased all-cause mortality in model 3 (HR: 1.36; 95% CI: 1.03–1.80).Smoothed curve fitting showed a U-shaped relationship between UHR and all-cause mortality, Previous epidemiologic studies have found significant differences in uric acid levels between males and females, with mean blood uric acid levels of approximately 5.0–6.0 mg/dL in males and 4.0–5.0 mg/dL in females, suggesting that uric acid is influenced by gender [42].Therefore, we further performed curve-fitting analysis stratified by gender, and found that the U-shaped relationship remained in male hypertensive patients, while a linear relationship was observed in females, which was attributed to the fact that male androgens (e.g., testosterone) may inhibit the synthesis of HDL and increase uric acid retention, forming a U-shaped curve [43], while female estrogens may up-regulate the level of HDL and promote uric acid excretion in females through up-regulation of uric acid-transporting proteins in the kidneys (such as URAT1, ABCG2) to promote uric acid excretion in females, thus attenuating the negative effect of UHR on mortality, showing a linear relationship [44].The threshold effect analysis showed that UHR in men was 0.14 when all-cause mortality was lowest. Furthermore, to the best of our knowledge, chronic inflammation has a major part in the development of hypertension [45,46], and Zenglei Zhang et al. have outlined that inflammatory vesicles (e.g., pyrin structural domain-containing proteins of NLR family NLRP1 and NLRP3) [47,48,49], inflammatory cell cytokines (IL-17, IL-6, TNF-α, IFN- γ, IL-1β) [50,51,52,53,54,55,56], and neuroinflammation [57,58,59,60] in the progression of essential hypertension [61], therefore, we explored the role of inflammation in the association between UHR and all-cause and cardiovascular mortality and found that neutrophils mediated 18.63%.

## Research strengths and limitations

A major strength of our study is that, first, it was a prospective cohort study that included hypertensive subjects from the nationally representative NHANES database, which reinforces the veracity and generalizability of our findings. Second, we adjusted for a series of covariates to determine the association between UHR and all-cause and cardiovascular mortality in hypertensive patients, which improved the accuracy of our study. In addition, we followed up our sample over time to improve the reliability of our study. Our study also emphasized the mediating role of inflammation in these associations. However, our study does have several limitations. First, our study was not comprehensive enough to assess the association of UHR between mortality from diabetes mellitus, other cause-specific mortality such as respiratory diseases, and mortality from different comorbidities of hypertension in hypertensive patients. Second, we performed multiple interpolation of missing data for variables, which, although it did not intrinsically affect the results of the study, may have led to a slight bias in the data for the variables. Third, as an observational study, the data analyzed were often subjective and failed to fully account for relevant confounding factors, thus additional research is required to validate our results. Finally, UHR data were obtained after only one measurement, which may affect the accuracy of UHR data.

## Conclusion

Our findings suggest a positive association between UHR and all-cause mortality in hypertensive patients, and we further stratified by gender to find a U-shaped relationship between UHR and all-cause mortality in male hypertensive patients, with men having the lowest all-cause mortality at a UHR level of 0.13, while assessing the critical mediating role of neutrophils between the two.
